# Advances in research on hyperoxia-induced acute lung injury: from pathogenesis to therapy

**DOI:** 10.3389/fcell.2026.1843981

**Published:** 2026-09-07

**Authors:** Yingcong Ren, Wujuan Xie, Kai Qi, Yingying Cai

**Affiliations:** 1 Chengdu Medical College The First Affiliated Hospital, Chengdu, China; 2 Xindu District People’s Hospital of Chengdu, Chengdu, China; 3 School of Basic Medical Sciences, Zunyi Medical University, Zunyi, China

**Keywords:** cell death, hyperoxia-induced acute lung injury, microRNAs, oxidative stress, stem cells

## Abstract

Hyperoxia-induced acute lung injury (HALI) is a serious complication of excessive oxygen exposure, yet its pathogenesis and treatment remain incompletely understood. A growing body of evidence suggests that HALI results from the interplay of oxidative stress, inflammation, immune dysregulation, organelle dysfunction, alveolar barrier disruption, and regulated cell death. In this review, we summarize recent advances in our understanding of these processes and discuss therapeutic strategies that have been investigated in experimental models. These approaches include antioxidant and anti-inflammatory agents, metabolic and mitochondrial modulators, interventions targeting iron metabolism and ferroptosis, barrier-protective therapies, and cell- or extracellular vesicle-based treatments. We also consider the limitations of the current evidence, particularly the differences between experimental models and clinical settings and the safety, delivery, and reproducibility challenges associated with emerging therapies. Further studies are needed to define the key regulatory mechanisms and temporal features of HALI, establish clinically relevant experimental models, and generate stronger clinical evidence to determine whether promising experimental interventions can be translated into effective therapies.

## 1 Introduction

Hyperoxia-induced acute lung injury (HALI) is acute pulmonary damage caused by oxygen toxicity during prolonged high-concentration oxygen exposure. Clinically, oxygen therapy is indispensable for critically ill patients, including those with acute respiratory distress syndrome or severe pneumonia. However, excessive oxygen can trigger oxidative stress and inflammatory cascades, ultimately inducing alveolar epithelial cells (AECs) apoptosis (Guo et al., 2024), ferroptosis (Li K. et al., 2024), and other forms of regulated cell death, impairing ventilation and gas exchange and worsening clinical outcomes. Balancing adequate oxygen delivery while minimizing hyperoxia-induced lung injury remains a key clinical challenge. Clinical and pathological observations have provided evidence that excessive oxygen exposure can cause clinically relevant pulmonary injury. Historical pathological studies demonstrated that prolonged exposure to high oxygen concentrations during mechanical ventilation was associated with pulmonary capillary leakage, alveolar epithelial and endothelial injury, pulmonary edema, hemorrhage, and, with longer exposure, fibroproliferative changes (Kapanci et al., 1972; Matsubara et al., 1986; Nash et al., 1967). Importantly, pulmonary capillary leakage may occur as early as 14 h after high-concentration oxygen exposure (Kapanci et al., 1972), indicating that oxygen-related pulmonary injury can develop relatively early. Clinical studies have further demonstrated that hyperoxemia is common during routine ICU care. In mechanically ventilated critically ill patients, the incidence of hyperoxemia increased from 15.6% to 22.4% during the first 48 h of treatment (Itagaki et al., 2015). Similarly, a retrospective multicenter cohort of 4,998 mechanically ventilated patients, 23.9% had arterial hyperoxemia despite a median FiO_2_ of 40%, and hyperoxemia was associated with increased ICU mortality (Schjørring et al., 2020). Other studies have linked hyperoxemia to prolonged ICU stay (Yamamoto et al., 2021), lower 30-day survival after cardiac arrest (Awad et al., 2023), and increased risks of acute brain injury and mortality during extracorporeal cardiopulmonary resuscitation (Shou et al., 2023). However, these associations are not consistent across all patient populations; for example, hyperoxemia was not significantly associated with 90-day mortality in some patients with sepsis (Catalisano et al., 2025). Together, these findings indicate that clinically relevant hyperoxemia may occur even during routine oxygen therapy and mechanical ventilation, highlighting the importance of appropriate oxygen management and the need to better understand the mechanisms underlying oxygen-induced lung injury.

At the mechanistic level, HALI represents a dynamic and interconnected process rather than a single pathological event. Excessive oxygen exposure disrupts pulmonary redox homeostasis and generates excessive ROS (Singer et al., 2021), which can initiate inflammatory responses (Bezerra et al., 2023), mitochondrial and endoplasmic reticulum dysfunction (Palma et al., 2024; Sidramagowda Patil et al., 2022), alveolar barrier disruption (Pao et al., 2021), and multiple forms of regulated cell death (Zhang and Armstrong, 2007; Endale et al., 2023). These processes are closely interconnected and may reinforce one another, forming a self-amplifying network that drives the progression of lung injury. Key signaling pathways, such as NF-κB (Fang et al., 2019), JAK/STAT (Li Z. et al., 2018), TGF-β and MAPK (Guo et al., 2022), have been implicated in this process, while emerging evidence has further highlighted the roles of ferroptosis, immune–epithelial interactions, and intercellular communication in HALI.

This complex pathogenesis has prompted the development of therapeutic strategies targeting different components of HALI. Current approaches include antioxidants (Jia et al., 2021), anti-inflammatory agents (Sitapara et al., 2020), metabolic and mitochondrial modulators (Chu et al., 2022; Patil et al., 2019), barrier-protective therapies (Pao et al., 2021), and cell or extracellular vesicle-based interventions (Wu Y. et al., 2021). Although many of these strategies have shown protective effects in experimental models, their mechanisms, therapeutic windows, and translational potential vary considerably. The complex interactions among pathological processes also raise the question of whether targeting a single pathway is sufficient to achieve effective and sustained protection.

In this review, we discuss the major molecular and cellular mechanisms underlying HALI, with emphasis on oxidative stress, inflammatory and immune responses, organelle dysfunction, alveolar barrier injury, and regulated cell death. We then review current preventive and therapeutic strategies according to their principal targets and mechanisms, covering pharmacological, metabolic, iron-related, cell-based, and extracellular vesicle-based approaches. Finally, we examine the limitations of current evidence and the major challenges for clinical translation, including differences between experimental models and patients, safety and delivery issues associated with emerging therapies, and the need for clinically relevant validation.

## 2 Molecular mechanisms of HALI

**FIGURE 1:**
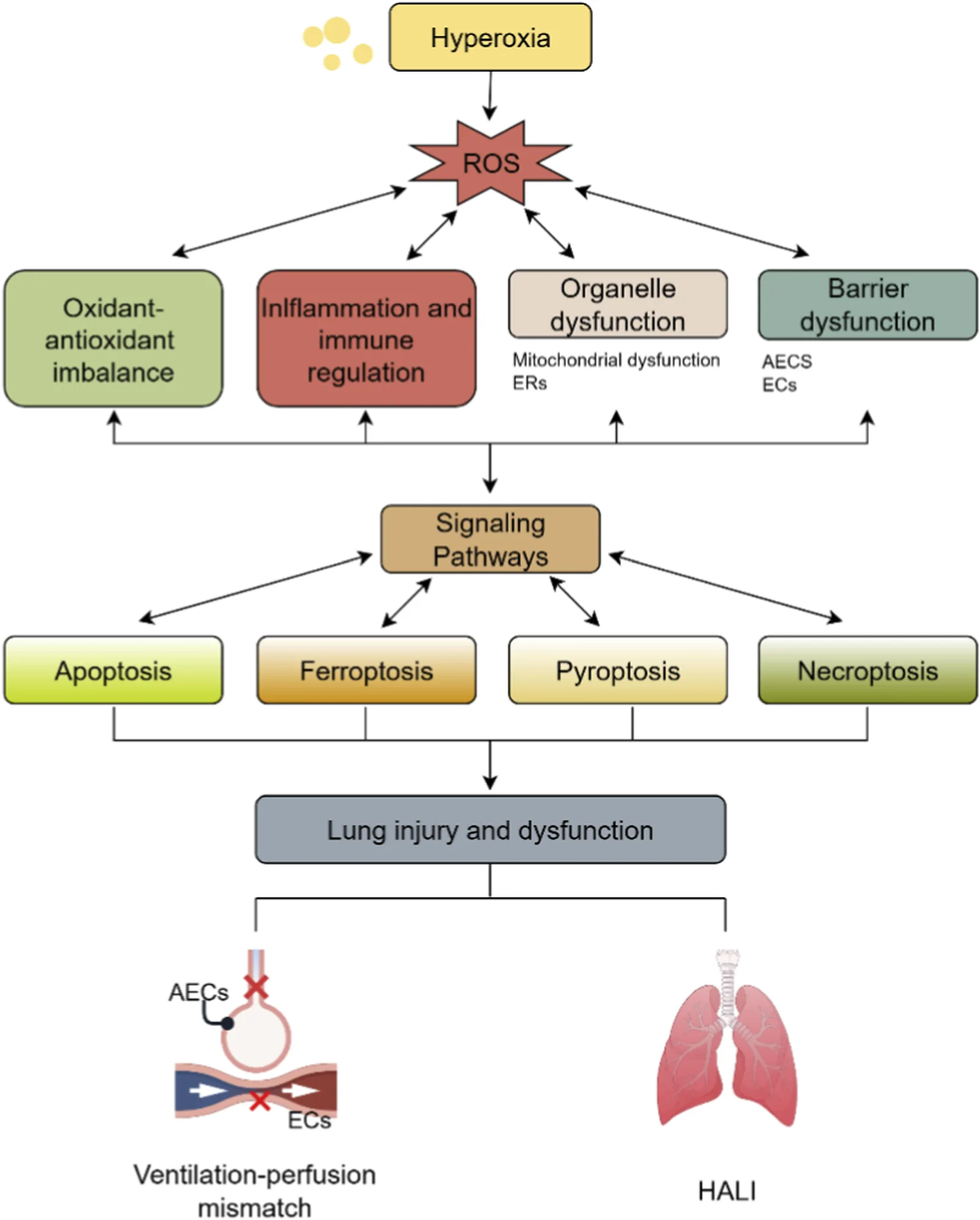
Pathogenic mechanisms of HALI. (Created with Figdraw).

The major pathogenic mechanisms underlying HALI are summarized in Figure 1. Hyperoxia triggers oxidative stress, inflammation, organelle dysfunction, barrier dysfunction and multiple forms of regulated cell death, ultimately leading to pulmonary injury.

### 2.1 Oxidant–antioxidant imbalance

Under hyperoxia, ROS accumulation is a central driver of HALI, mediating cellular and tissue injury (Singer et al., 2021). Major ROS sources include NADPH oxidases, mitochondrial electron transport, and dysregulated nitric oxide synthase activity (Dryden, 2018). Among NADPH oxidases (NOX), NOX3 and NOX4 aggravate lung injury through distinct mechanisms: NOX3 promotes oxidative damage and endothelial dysfunction, which can be attenuated through the HSP70-TLR4 axis (Zhang Y. et al., 2016), whereas NOX4 increases mitochondrial ROS and triggers AECs apoptosis (Harijith et al., 2022). Both contribute to superoxide production and tissue damage.

Antioxidant defenses normally counteract ROS, with nuclear factor erythroid 2-related factor 2 (NRF2) acting as a key transcriptional regulator. Under basal conditions, NRF2 is sequestered in the cytoplasm by Kelch-like ECH-associated protein 1 (Li et al., 2022). Oxidative stress disrupts this interaction, allowing NRF2 nuclear translocation and activation of antioxidant response elements, inducing enzymes such as HO-1, SOD, catalase (CAT), glutathione peroxidase (GPX), and glutathione reductase (GR) (Ngo and Duennwald, 2022; Cyran and Zhitkovich, 2022). Chronic hyperoxia, however, suppresses NRF2 through altered nucleocytoplasmic transport or pathway crosstalk, reducing antioxidant enzyme expression and impairing ROS clearance (Amata et al., 2017; Yang et al., 2023). NRF2 deficiency worsens lung injury and delays recovery by limiting AECs and endothelial regeneration and prolonging immune infiltration (Reddy et al., 2009). In contrast, NRF2 activation alleviates oxidative stress and inflammation and promotes pulmonary repair.

Other oxidative metabolism enzymes also regulate ROS and inflammation (Magliocco et al., 2019). Cytochrome P450 (CYP450) isoforms show differential effects: CYP1B1 deletion mitigates hyperoxic lung injury (Veith et al., 2018), whereas CYP1A1 or CYP1A2 deficiency increases susceptibility (Lingappan et al., 2017; Wang et al., 2015), suggesting isoform-specific roles. Sirtuins SIRT1 and SIRT3, two NAD^+^-dependent deacetylases, also participate in the modulation of oxidative stress (Zhao et al., 2020). SIRT1 inhibition reduces AECs apoptosis (Potteti et al., 2016), while SIRT3 enhances manganese superoxide dismutase activity and protects against oxidative injury (Tian and Zhang, 2018)^.^

Collectively, excessive ROS production and impaired antioxidant defenses form a self-amplifying cycle that drives HALI progression. Strategies that restore NRF2 activity (Wan et al., 2020) or enhance superoxide scavenging may alleviate hyperoxia-induced lung injury, although the roles of additional oxidative metabolism-related enzymes require further investigation.

### 2.2 Inflammation and immune dysregulation

Under hyperoxic conditions, disruption of cellular redox balance and excessive ROS damage DNA, proteins, and lipids. DNA strand breaks induce mutations and promote apoptosis or necrosis (Suryadevara et al., 2019). Protein oxidation causes enzyme inactivation and dysfunction (Guo et al., 2021), while lipid oxidation leads to lipid peroxidation, damaging membrane integrity and even inducing ferroptosis (Wu et al., 2024). Beyond direct cytotoxicity, oxidative stress also triggers inflammatory responses and pro-inflammatory cytokine release, thereby accelerating lung injury (Bezerra et al., 2023).

Hyperoxia activates the classical NF-κB pathway, inducing cytokines such as TNF-α, IL-6, and IL-1β and promoting inflammatory infiltration (Yen et al., 2020). Apoptosis signal-regulating kinase 1 (ASK1), a p38MAPK serine/threonine kinase activated by redox imbalance and Ca^2+^ signaling, is another key regulator (Toyama et al., 2021). Under hyperoxia, ASK1 promotes AECs inflammation and apoptosis through the MAPK pathway, whereas ASK1 knockout reduces lung inflammation, immune cell infiltration, and apoptosis (Fukumoto et al., 2016).

The NOD-like receptor family pyrin domain-containing 3 (NLRP3) inflammasome and toll-like receptors (TLRs) are also activated under hyperoxia. NLRP3 senses oxidative stress and cellular injury, promoting inflammasome assembly and IL-1β maturation, thereby aggravating lung injury (Zhong et al., 2013; Fukumoto et al., 2013). NLRP3-deficient mice show reduced inflammation and AECs apoptosis after hyperoxic exposure, although mortality is paradoxically increased, suggesting a complex role in HALI (Hong et al., 2019; Mizushina et al., 2015). TLRs, receptors for damage-associated molecular patterns (DAMPs), are widely expressed in macrophages, dendritic cells, and AECs and recognize pathogen-derived molecules and endogenous stress signals (Leventhal and Schröppel, 2012; Kawai and Akira, 2010). Hyperoxia-induced activation of TLR3/7 enhances downstream inflammatory signaling and cytokine release. Deletion of TLR3/7 alleviates hyperoxia-induced lung injury and improves lung function (Murray et al., 2008; Zheng et al., 2020a).

High-mobility group box 1 (HMGB1), a prototypical DAMP released after cellular injury, binds to TLRs and activates inflammatory signaling in HALI. Hyperoxia-induced NF-κB activation promotes HMGB1 release, while extracellular HMGB1 impairs macrophage immune defense (Gauthie et al., 2023). Reducing HMGB1 release decreases cytokine infiltration and restores Alveolar macrophages (AMs) phagocytosis, thereby improving pathogen clearance and attenuating lung injury (Yimam et al., 2023; Patel et al., 2020). AMs regulate inflammation through polarization. M1 macrophages produce pro-inflammatory cytokines and ROS (Xia et al., 2023), whereas M2 macrophages release anti-inflammatory cytokines and growth factors that promote tissue repair (Kim and Nair, 2019). Hyperoxia shifts macrophages toward the M1 phenotype and impairs phagocytosis, sustaining inflammation (Guo et al., 2021; Zhu et al., 2025). Activation of the ATP-gated purinergic receptor P2X7 further promotes IL-1β release, increases AECs permeability, and aggravates pulmonary edema (Galam et al., 2016).

Neutrophil infiltration is another contributor to pulmonary inflammation in HALI. C-X-C motif chemokine receptor 1 (CXCR1), a G protein-coupled receptor highly expressed in immune cells, mediates neutrophil recruitment mainly through IL-8 binding (Mattos et al., 2020; Alsabani et al., 2022). In HALI models, CXCR1 knockout reduces neutrophil infiltration and ROS release. CXCR1 deletion in pulmonary endothelial cells also enhances ROS clearance and regulates glutamine metabolism, attenuating HALI (Cesta et al., 2021; Wang X. et al., 2020; Qin H. et al., 2023).

Hyperoxia-induced cytokine release may also trigger systemic inflammatory responses through circulation, which may explain elevated peripheral inflammatory cytokines and reduced protective alveolar proteins in patients with hyperoxemia ().

Overall, oxidative stress promotes HALI progression by activating multiple inflammatory pathways. TLRs activation, NLRP3 inflammasome assembly, macrophage polarization, and neutrophil infiltration collectively drive hyperoxia-induced pulmonary inflammation and lung injury.

### 2.3 Organelle dysfunction

Mitochondria serve as the cell’s primary energy generators, governing energy metabolism, redox homeostasis, signaling, and apoptosis. In the mitochondrial matrix, pyruvate and fatty acids are oxidized to produce NADH, which donates electrons to the electron transport chain (ETC.) on the inner membrane. This process powers oxidative phosphorylation and ATP synthesis (Okoye et al., 2023). Mitochondria also represent a major source of ROS; electron leakage from the ETC generates superoxide, hydrogen peroxide, and hydroxyl radicals, promoting oxidative stress, inflammation, metabolic dysfunction, and cell death (Palma et al., 2024).

Uncoupling protein 2 (UCP2), a proton transporter in the inner mitochondrial membrane, uncouples oxidative phosphorylation, lowering membrane potential and ATP yield while reducing ROS production. Preserving UCP2 expression helps mitigate lung injury (Raghavan et al., 2022).

NOD-like receptor family member X1 (NLRX1), situated on the outer mitochondrial membrane, modulates mitochondrial function, ROS generation, autophagy, and apoptosis (Nagai-Singer et al., 2019). In hyperoxia-exposed mice, NLRX1 levels rise significantly. NLRX1 deletion attenuates MAPK signaling, decreases inflammatory cell infiltration and protein extravasation, and alleviates lung injury by suppressing mitochondrial apoptosis and AECs death (Kim et al., 2023).

Mitofusins (MFN1/2), outer membrane GTPases, mediate mitochondrial fusion and maintain functional balance. Hyperoxia impairs MFN activity, causing mitochondrial fragmentation, disrupted energy metabolism, apoptosis, and lung damage (Mu et al., 2020). PTEN-induced kinase 1 (PINK1) is essential for mitochondrial quality control and mitophagy (Tian et al., 2022). Normally, PINK1 is imported and degraded; oxidative stress dissipates membrane potential, leading to PINK1 accumulation on the outer membrane and initiation of mitophagy to clear damaged mitochondria (Han et al., 2023). Thyroid hormone T3 pretreatment protects against HALI by upregulating mitochondrial biogenesis, fusion, and PINK1-dependent mitophagy, thereby improving mitochondrial function and reducing apoptosis (Zhang et al., 2021).

Additional regulators influence mitochondrial dynamics and quality control. B lymphoma Mo-MLV insertion region 1 (BMI1), a polycomb group protein, controls cell proliferation and survival. Hyperoxia downregulates BMI1, which normally represses MFN, PINK1, and Drp1, thereby modulating mitochondrial fusion/fission, mitophagy, and metabolism. BMI1 reduction exacerbates cellular injury and death (Hernández-Cuervo et al., 2022). A-kinase anchoring protein 1 (AKAP1), another outer membrane protein, similarly supports mitochondrial homeostasis. In HALI, AKAP1 deficiency aggravates pulmonary inflammation, elevates PINK1, promotes excessive mitophagy and morphological abnormalities in AECs, and worsens injury (Narala et al., 2018). AKAP1 loss also decreases Drp1 phosphorylation at Ser637, disrupting mitochondrial dynamics, impairing TCA cycle enzyme activity ETC function, elevating pro-apoptotic proteins, and contributing to HALI progression (Soundararajan et al., 2022).

Mitochondria and the Endoplasmic reticulum (ER) are closely interconnected, and hyperoxia disrupts ER homeostasis in addition to mitochondrial function (Jiang et al., 2024). Excessive ROS, inflammation, and protein misfolding contribute to hyperoxia-induced Endoplasmic reticulum stress (ERs). For example, AKAP1 downregulation aggravates lung injury by promoting both mitochondrial dysfunction and ERs (Sidramagowda Patil et al., 2022). Hyperoxia alters ER membrane proteins, including the Sigma-1 receptor (Sig-1R), caspase-3, and CCAAT/enhancer-binding protein homologous protein. Sig-1R, an ER-resident chaperone, modulates ERs, and its activation suppresses hyperoxia-induced apoptosis, mitigating lung injury (Huang et al., 2024). Family with sequence similarity 134 member B (FAM134 B), also known as ER-phagy regulator 1, is critical for ER-phagy and cellular homeostasis. FAM134 B activation enhances ER-phagy, preserves ER function, reduces AECs apoptosis and epithelial–mesenchymal transition, and alleviates HALI (Guo et al., 2024; González et al., 2023).

SIRT1, a redox-regulating deacetylase, also modulates ERs. SIRT1 overexpression suppresses ERs signaling and attenuates lung injury, underscoring its multifaceted role in HALI pathogenesis (Sun et al., 2017).

### 2.4 Barrier dysfunction

Hyperoxia directly disrupts AECs and endothelial cell (ECs) barrier integrity, increasing alveolar permeability, compromising structure, and exacerbating pulmonary edema and protein-rich fluid extravasation.

In AECs, tight junction proteins, including claudin-3, -4, and -18, are critical for barrier function and fluid clearance. Hyperoxia downregulates these proteins, promoting edema, whereas their upregulation alleviates lung injury (Pao et al., 2021; Shen et al., 2021). TWIK-related potassium channels (TREK-1/2) stabilize membrane potential, prevent hyperoxia-induced depolarization, and reduce intracellular calcium, thereby attenuating calcium-dependent pro-inflammatory signaling and AECs injury. TREK1/2 deficiency further impairs surfactant protein A (SP-A) and pro–surfactant protein C(SP-C) expression, worsening lung damage (Zyrianova et al., 2020; Schwingshackl et al., 2017).

In ECs, vascular endothelial growth factor D (VEGF-D) activates VEGFR-2/3, enhancing proliferation, migration, angiogenesis, and vascular permeability. VEGF-D knockout reduces edema in HALI models (Bates, 2010; Sato et al., 2016). The angiotensin II type 2 receptor (AT_2_R), predominantly expressed in alveolar fibroblasts, exerts anti-inflammatory and cytoprotective effects. AT_2_R deficiency under hyperoxia impairs barrier function, increases oxidative stress, thickens alveolar septa, and elevates mortality, highlighting its role in maintaining alveolar–capillary integrity (Wang et al., 2017; Abadir et al., 2024).

Overall, reinforcing AECs and ECs barrier function represents a promising strategy to mitigate pulmonary edema and protein leakage in HALI.

### 2.5 Signaling pathways

Oxidative–antioxidative imbalance, inflammatory responses, immune activation, and organelle dysfunction in HALI are closely linked to downstream signaling cascades that mediate amplification and network regulation. Several key signaling pathways have therefore been implicated in the pathogenesis of HALI.

#### 2.5.1 NF-κB pathway

NF-κB is a family of transcription factors broadly expressed in mammalian cells that regulate gene trans cription, cytokine production, and cell survival, enabling cellular responses to diverse stimuli (Capece et al., 2022). As a key regulator of innate and adaptive immunity (Wu G. et al., 2021), NF-κB activation in HALI enhances inflammatory signaling and promotes the release of pro-inflammatory mediators. Suppression of this pathway reduces cytokine production and alleviates lung injury, highlighting NF-κB as a potential therapeutic target for hyperoxia-induced inflammation (Tung et al., 2021; Chen CM. et al., 2018).

#### 2.5.2 TGF-β/smad pathway

TGF-β signaling controls cell fate through receptor-regulated Smad proteins that translocate to the nucleus and regulate gene transcription. Abnormal activation of this pathway has been linked to multiple pathological conditions, including fibrosis, immune disorders, and cancer (Yu et al., 2024; Wang et al., 2025). In hyperoxia-enhanced ventilator-induced lung injury, Src-dependent Smad3 activation drives neutrophil infiltration, inflammatory mediator release, and increased microvascular permeability. Accordingly, blockade of TGF-β/Smad signaling can attenuate inflammatory lung injury (Li LF. et al., 2015).

#### 2.5.3 JAK/STAT3 signaling pathway

The JAK/STAT3 cascade functions as an important intracellular pathway controlling cell proliferation, survival, and inflammatory responses. Following receptor activation, Janus kinases phosphorylate STAT3, which subsequently translocates to the nucleus to regulate target gene transcription. Under hyperoxic conditions, excessive ROS activates JAK/STAT3 signaling in HALI, promoting AECs apoptosis (Qin S. et al., 2023). Activation of this pathway also increases the expression of inflammatory cytokines such as IL-6 and TNF-α, thereby amplifying inflammatory responses (Li Z. et al., 2018).

#### 2.5.4 p38 MAPK pathway

P38 MAPK is a stress-responsive signaling pathway that mediates cellular responses to stimuli such as cytokines, ultraviolet radiation, and oxidative stress, and participates in the regulation of apoptosis, differentiation, and autophagy. Hyperoxia can trigger p38 MAPK activation and contribute to the progression of lung injury. Pharmacological inhibition of p38 MAPK mitigates IL-17 A–induced ferroptosis in AECs (Guo et al., 2022), preserves mitochondrial function and reduces oxidative stress–related damage (Raghavan et al., 2022), and restores epithelial barrier integrity by promoting Claudin-18 expression (Shen et al., 2021), thereby alleviating HALI.

#### 2.5.5 PI3K/AKT pathway

PI3K/AKT pathway typically begins with stimulation of receptor tyrosine kinases or G protein–coupled receptors, leading to PI3K activation and conversion of phosphatidylinositol-4,5-bisphosphate to phosphatidylinositol-3,4,5-trisphosphate (PIP3) . PIP3 subsequently recruits and activates AKT, which regulates multiple downstream targets involved in cell survival and metabolism (He et al., 2021). In HALI, PI3K/AKT signaling exhibits dual effects. Activation via NRF2 stimulation or PTEN inhibition reduces oxidative stress and apoptosis, exerting protective effects (Reddy et al., 2015; Qin et al., 2019). However, excessive activation may also enhance inflammatory responses (Reddy et al., 2015). Therefore, therapeutic targeting of PI3K/AKT requires consideration of its context-dependent roles during different stages of HALI.

### 2.6 Regulated cell death

Beyond oxidative damage and inflammation, cell death is a central event in HALI. The diversity of cell death modalities not only reflects tissue injury severity but also shapes local and systemic immune responses, inflammation, and repair. Therapeutic strategies aim to limit oxidative injury and inflammation while promoting cell survival, thereby reducing AECs loss. Current literature identifies apoptosis, ferroptosis, pyroptosis, and necroptosis as key contributors, each with distinct mechanisms. These modalities may interact or act synergistically depending on cell type and injury stage, highlighting the complexity of cell fate regulation in HALI.

#### 2.6.1 Apoptosis

Apoptosis, the primary form of programmed cell death in HALI, is largely mediated by mitochondria-dependent pathways. Excessive ROS damage mitochondrial lipids ETC complexes, collapsing membrane potential and triggering cytochrome c release. Cytochrome c binds Apaf-1, activating Caspase-9 and subsequently effector Caspase-3/7, culminating in DNA fragmentation (Chen et al., 2003). Dysregulation of Bcl-2 family proteins, including pro-apoptotic Bax and anti-apoptotic Bcl-2, is central (Susnow et al., 2009). Hyperoxia-induced ROS oxidatively modify Bcl-2, impairing its function while promoting Bax oligomerization and mitochondrial pore formation (Zhang and Armstrong, 2007). Conditional knockout of Bax/Bak in AECs markedly reduces apoptosis, alleviates lung injury, and prolongs survival (Budinger et al., 2011).

ROS not only act as triggers of apoptosis but can also exacerbate lung injury through a vicious “oxidative stress–inflammation–apoptosis” cycle. Mitochondrial ROS also amplify apoptosis via JNK signaling (Makena et al., 2011). NLRP3-deficient mice exhibit reduced Caspase-3 activation, attenuated NF-κB signaling, decreased inflammatory infiltration, and lower histopathological injury (Fukumoto et al., 2013). Apoptotic cells release mitochondrial DNA, activating AMs through TLR9, which promotes IL-6 and TNF-α secretion, further exacerbating injury (Lin et al., 2018).

#### 2.6.2 Ferroptosis

Ferroptosis is an iron-dependent, lipid peroxidation-driven cell death. Hyperoxia-induced ROS attack polyunsaturated fatty acids, initiating lipid peroxidation (Endale et al., 2023). ROS inhibit the cystine/glutamate antiporter, deplete glutathione, and impair GPX4 activity, disrupting mitochondrial cristae and plasma membrane integrity. Concurrently, increased transferrin receptor expression and reduced ferritin heavy chain elevate labile Fe^2+^, catalyzing hydroxyl radical formation via the Fenton reaction, creating a self-amplifying “lipid peroxidation–iron overload” loop (Xing et al., 2023; Li Q. et al., 2024). Ferroptosis occurs in AECs during HALI and its inhibition improves lung injury (Li K. et al., 2024; Zhao et al., 2025), though mechanistic studies remain limited.

#### 2.6.3 Pyroptosis and necroptosis

Pyroptosis, an inflammatory programmed cell death, is mediated by the NLRP3 GSDMD axis. ROS and other stimuli activate NLRP3, which interacts with NIMA-related kinase 7 and forms the NLRP3–ASC–pro-Caspase-1 complex. Activated Caspase-1 cleaves GSDMD, forming membrane pores and releasing IL-1β and IL-18 (Zheng et al., 2020b; Kelley et al., 2019). While studies in HALI are limited, neonatal hyperoxia models show GSDMD knockout reduces macrophage infiltration and improves alveolarization (Sonny et al., 2023). Pyroptosis likely contributes to early inflammatory responses in HALI, though further validation is needed.

Necroptosis, mediated via RIPK1/RIPK3–MLKL signaling, can also be triggered by ROS in AECs (Yuan et al., 2025). Evidence from COPD models shows cigarette smoke–induced ROS promotes RIPK3/MLKL phosphorylation and necroptosis, while antioxidants or necroptosis inhibitors reduce injury (Chen D. et al., 2021; Luan et al., 2022). By analogy, hyperoxia-induced oxidative stress may similarly drive necroptosis in HALI.

Mitochondrial dysfunction underlies these processes, with excessive ROS serving as a common upstream trigger. HALI may involve additional cell death modalities, such as cuproptosis. Different modalities may predominate at distinct stages and interact synergistically, complicating tissue repair and recovery (The major mechanisms, molecular regulators, signaling pathways and pathological consequences involved in HALI are summarized in Table 1)

**TABLE 1:** Major mechanisms, molecular regulators, signaling pathways and consequences involved in HALI.

Mechanisms		Targets/regulators	Signaling pathways	Major consequences
Oxidant–antioxidant imbalance	Oxidative stress	↑NOX3, NOX4, CYP1B1, SIRT1; ↓CYP1A1/2, SIRT3	↑JAK/STAT3, PI3K/AKT	↑ROS accumulation, oxidative damage
	Antioxidant impairment	↓NRF2, HO-1, SOD, CAT, GPX, GR		
Inflammation and immune dysregulation	Inflammation	↑ASK1, NLRP3, TLR3/7, HMGB1	↑NF-κB, p38MAPK, TGF-β/Smad	↑TNF-α, IL-6, IL-1β cytokine release, immune infiltration
	Immune dysregulation	↑P2X7, macrophage M1, CXCR1		↑Macrophage activation, neutrophil recruitment
Organelle dysfunction	Mitochondrial dysfunction	↓UCP2, BMI1,AKAP1; ↑NLRX1, MFN1/2; ↑PINK1	↑MAPK	↓MMP, ATP; ↑mtROS, apoptosis
	ERs	↓AKAP1, Sig-1R, FAM134B, SIRT1	—	↓ER autophagy/homeostasis; ↑ERs, apoptosis
Barrier dysfunction	AECs	↓Claudin-3/4/18, TREK-1/2	↑p38 MAPK TGF-β/Smad	↓SP-A/C; ↑permeability, edema
	ECs	↑VEGF-D; ↓AT_2_R		
Regulated cell death	Apoptosis	↓Bcl-2; ↑Bax, cyt c, Apaf-1, caspase-3/7/9	↑MAPK; NF-κB; JAK/STAT3; PI3K/AKT	↑apoptotic body formation
	Ferroptosis	↓GPX4, FTH1, GSH; ↑TFRC, Fe^2+^	↑p38 MAPK	↑Lipid peroxidation
	Pyroptosis	↑NLRP3; Caspase-1, GSDMD	↑NF-κB	↑Inflammatory cell death
	Necroptosis	↑RIPK1/3, MLKL	—	↑Membrane rupture

## 3 Strategies for the prevention and treatment of HALI

### 3.1 Oxidative stress regulation

Enhancing endogenous antioxidant defenses is a major approach to counteracting hyperoxia-induced oxidative injury. Compounds such as etomidate, lycium barbarum polysaccharides, wedelolactone, and sulforaphane activate NRF2, a key regulator of cellular antioxidant responses, to mitigate lung injury. Etomidate alleviates oxidative stress and inflammation via the NRF2/HO-1 pathway (Jia et al., 2021); Lycium barbarum polysaccharides enhance antioxidant enzyme activity through NRF2 activation (Zheng et al., 2019); Wedelolactone inhibits ferroptosis in AECs via NRF2/HO-1, reducing lung damage (Li K. et al., 2024); and sulforaphane promotes transcription of mitochondrial energy metabolism–related genes via NRF2, improving lung injury (Cho et al., 2019). Multienzyme-active melanin nanodots provide a recent multi-target approach by directly scavenging ROS and activating the NRF2/HO-1 pathway, while modulating macrophage responses to reduce oxidative stress, inflammation, and apoptosis in experimental HALI (Sun et al., 2025). In contrast, Soluble epoxide hydrolase (sEH), an enzyme that protective epoxides, can diminish antioxidant effects. sEH knockout mice show NRF2 a degrades activation, increased HO-1 and SOD activity, and reduced ROS levels (Li PS. et al., 2018; Liu et al., 2018). These findings highlight NRF2 as a central mediator of antioxidant protection in HALI.

CYP1A induction may provide another means of reducing oxidative injury. β-Naphthoflavone (BNF) induces CYP1A1 and CYP1A2 and enhances xenobiotic metabolism and detoxification (Park et al., 2019). In experimental HALI, BNF reduces oxidative stress and inflammation, suggesting that CYP1A-mediated metabolic regulation may contribute to lung protection (Callaway et al., 2020). However, the specific substrates and downstream mechanisms involved remain unclear.

Iron metabolism is also closely linked to oxidative injury and ferroptosis. Lactoferrin reduces ROS production and inflammation and alleviates hyperoxia-induced lung and kidney injury (Yen et al., 2020). Deferoxamine (DFO), an iron chelator, promotes AECs repair, reduces alveolar exudation, and improves pulmonary function in hyperoxia and mechanical ventilation models (Wang X. X. et al., 2020). These effects may be related to reduced iron-dependent ROS generation and ferroptosis. More recently, nicotinamide mononucleotide (NMN) was reported to attenuate hyperoxia-aggravated septic lung injury by enhancing GPX4-mediated anti-ferroptotic signaling in AECs (Zhao et al., 2025) Together, these findings suggest that targeting iron availability and antioxidant defenses may help limit ferroptosis, although the evidence for NMN is currently derived from a septic lung injury model.

### 3.2 Anti-inflammatory and immunomodulatory strategies

Excessive inflammation driven by oxidative stress is a central feature of HALI. Various anti-inflammatory agents protect the lung by modulating distinct inflammatory pathways and cell death modalities. HMGB1 has been identified as a core mediator: ascorbic acid, sulforaphane (Patel et al., 2020), the α7 nicotinic acetylcholine receptor agonist GTS-21 (Sitapara et al., 2020), probucol (Kawaguchi et al., 2017), and astragaloside (Chen Y. et al., 2021) reduce oxidative stress–induced HMGB1 release, thereby limiting macrophage activation and alleviating lung injury.

Targeting the NF-κB pathway and downstream cytokines such as IL-17 A represents another therapeutic avenue. Aspirin activates the pro-resolving mediator resolvin D1, suppressing NF-κB nuclear translocation and reducing TNF-α and IL-6 release (Tung et al., 2021; Cox et al., 2015). Salidroside specifically inhibits IL-17 A–induced ferroptosis in AECs via the p38 MAPK pathway (Guo et al., 2022). Other approaches modulate inflammatory metabolism: simvastatin enhances endogenous nitric oxide (NO) to lower cytokine levels (Bao et al., 2018), whereas roflumilast raises cAMP by inhibiting phosphodiesterase-4 (PDE4), reducing neutrophil infiltration (Cao et al., 2020).

Targeting endogenous inflammatory mediators represents a direct approach to mitigate HALI. IL-3 deficiency significantly reduces hyperoxia-induced TNF-α and IL-6 expression in lung tissue and attenuates lung injury in mice (Huang et al., 2018). Likewise, suppressor of cytokine signaling-1(SOCS-1), a negative regulator of IL-6 signaling, decreases lipid peroxidation and inflammatory cell infiltration while improving survival under hyperoxic conditions (Galam et al., 2015). In addition, supplementation with exogenous anti-inflammatory cytokines such as IL-10 enhances compensatory anti-inflammatory responses and alleviates pulmonary inflammation and edema (Li H. et al., 2015). Given the multi-target nature of inflammatory regulation in HALI, therapeutic strategies combining inhibition of endogenous pro-inflammatory factors with supplementation of exogenous anti-inflammatory mediators may provide more effective protection against lung injury.

### 3.3 Metabolic and gut–lung axis regulation

Metabolic modulators in HALI are less studied, but interventions such as high-fiber diets and acetate supplementation show protective effects. These strategies mainly act by reshaping the gut microbiota and increasing anti-inflammatory metabolites. Specifically, they enhance short-chain fatty acid production, including acetate, which may protect the lung via the gut–lung axis by suppressing inflammatory mediator release and reducing AECs injury. Additionally, these metabolites exhibit antioxidant activity, mitigating oxidative stress and preserving pulmonary function (Chu et al., 2022). Despite these findings, the precise mechanisms remain to be fully elucidated.

### 3.4 Mitochondrial function modulators

Hyperoxia-induced mitochondrial dysfunction disrupts energy metabolism, depolarizes mitochondrial membrane potential (MMP), and promotes excessive ROS release, ultimately triggering cell death. Maintaining mitochondrial integrity is therefore critical for lung protection. Recent studies show that multiple modulators preserve mitochondrial homeostasis via diverse mechanisms. Alda-1 enhances ALDH2 activity, reducing ROS accumulation, preventing mitochondrial membrane collapse, and maintaining energy metabolism in pulmonary ECs (Patil et al., 2019). Mitoquinone (MitoQ), a mitochondria-targeted antioxidant, transfers electrons, suppresses ROS, and restores membrane potential, stabilizing mitochondrial function and cellular energy (Audi et al., 2022). Nitro-fatty acids, such as 10-nitro-oleic acid, covalently modify mitochondrial proteins, suppress ROS, and enhance antioxidant enzyme activity (Narala et al., 2022). Endothelial-derived stanniocalcin-1 improves mitochondrial oxidative phosphorylation via TLRs signaling, maintaining bioenergetic supply and preventing hyperoxia-induced metabolic failure (Zhang et al., 2019).

Several modulators also inhibit mitochondrial apoptosis in AECs. Resveratrol reduces mitochondrial DNA oxidative damage and pore opening via SIRT1 activation, decreasing cytochrome c release and caspase-3–mediated apoptosis (Zhu et al., 2021). Thioredoxin activates PGC-1α through p38 MAPK, promoting mitochondrial biogenesis and respiratory chain protein expression, while preserving UCP2 to limit ROS production and cell death (Raghavan et al., 2022). Collectively, these interventions act through antioxidant activity, structural repair, metabolic reprogramming, and apoptosis inhibition, forming a coordinated network that underpins mitochondria-targeted therapeutic strategies.

### 3.5 Alveolar barrier protectants

Enhancing alveolar barrier integrity is a key strategy for mitigating HALI. 4-Phenylbutyric acid (4-PBA) improves AECs barrier function by suppressing ERs, upregulating Claudin-4, and reducing pulmonary edema and inflammation. 4-PBA also inhibits ERs–associated proteins and NF-κB activation, decreasing AECs apoptosis. Notably, Claudin-4 knockdown diminishes 4-PBA’s protective effects, highlighting its central role (Pao et al., 2021).

Combination strategies further enhance barrier protection. The synthetic surfactant B-YL combined with the PPARγ agonist pioglitazone reduces HALI by inhibiting Wnt and TGF-β signaling, suppressing inflammatory mediator release, and decreasing apoptosis, while B-YL restores surfactant activity and alveolar stability (Kurihara et al., 2023). Similarly, in a rat “two-hit” model of LPS and hyperoxia, intravenous N-acetylcysteine plus intratracheal exogenous surfactant synergistically enhanced antioxidant capacity, suppressed IL-6, TNF-α, and IL-1β release, and significantly reduced pulmonary edema and leukocyte infiltration, outperforming single-agent treatments (Kolomaznik et al., 2023).

These findings indicate that barrier-protective agents, particularly when combined with antioxidants and anti-inflammatory therapies, represent an effective adjunctive approach for HALI management.

### 3.6 Gas molecule therapy

Gaseous signaling molecules such as hydrogen and inhaled NO have demonstrated protective effects in HALI. Hydrogen mitigates HALI by upregulating SIRT1, alleviating ERs, and reducing pulmonary edema and AECs apoptosis (Sun et al., 2017; Lu et al., 2018). Inhaled NO attenuates lung damage by suppressing plasminogen activator inhibitor-1(PAI-1) expression, thereby reducing fibrin deposition (Chen et al., 2015). These gasotransmitters act via distinct mechanisms, suggesting that supplementing oxygen therapy with small proportions of hydrogen or NO may provide additional lung protection. Nevertheless, further preclinical and clinical studies are needed to confirm their safety and efficacy.

### 3.7 Cell and molecular therapy

Cell and molecule based therapies have emerged as potential approaches for promoting lung repair and modulating inflammation in HALI. Induced pluripotent stem cells (iPSCs) possess broad differentiation potential, enabling tissue repair and functional restoration. In HALI models, iPSCs modulate inflammatory responses and oxidative stress while promoting lung tissue and diaphragmatic repair (Gazdhar et al., 2018). In hyperoxia-enhanced mechanical ventilation–induced diaphragmatic dysfunction, iPSCs improve diaphragmatic function by inhibiting Src tyrosine kinase and forkhead box O1(FOXO1), thereby reducing oxidative stress and inflammation (Li et al., 2016).

Stem cell–derived alveolar-like macrophages (ALMs) also show strong therapeutic potential. ALMs exhibit robust survival and immune stability under hyperoxic conditions, maintaining proliferative capacity and antibacterial activity at 60% oxygen and surviving in the lung even at 90% oxygen. Under hyperoxia, ALMs retain the ability to phagocytose *Pseudomonas aeruginosa* and reduce CD11 b^+^ inflammatory cell accumulation in LPS-induced pneumonia models, suggesting a role in preserving pulmonary immune homeostasis and antimicrobial defense (Litman et al., 2024).

Overall, stem cell–based therapies represent a biological approach distinct from small-molecule interventions, providing lung protection through modulation of inflammation, oxidative stress, and immune responses. Their differentiation capacity and broad regulatory effects highlight promising translational potential for HALI treatment. However, their clinical application remains challenging because of concerns regarding cell survival and engraftment, tumorigenicity, aberrant differentiation, and long-term safety.

Cell-free approaches based on extracellular vesicles (EVs) may overcome some limitations of direct cell transplantation. EVs mediate intercellular communication by delivering bioactive molecules and may also serve as biomarkers of lung injury (Higashi et al., 2002). Among EVs cargoes, miRNAs are the most extensively studied regulators in HALI. Several miRNAs exert protective effects. For instance, miR-20 b alleviates HALI by targeting mitochondrial fusion proteins MFN1 and MFN2, thereby inhibiting mitochondria-mediated apoptosis (Mu et al., 2020). miR-21–5p protects AECs via activation of the PTEN/AKT pathway (Qin et al., 2019)^,^ while miR-16 reduces AECs apoptosis by regulating the TGF-β/Smad2 and JAK/STAT3 signaling pathways (Li Z. et al., 2018). In contrast, miR-185 promotes oxidative stress–induced AECs death and may serve as a biomarker of pulmonary injury (Zhang D. et al., 2016). Importantly, EV-mediated effects are context-dependent. AECs-derived EVs can aggravate inflammation by activating the ROCK1 pathway in AMs or delivering pro-inflammatory miRNAs (Moon et al., 2015; Israeli et al., 1997). Whereas mesenchymal stem cell–derived exosomes exert therapeutic effects by transferring protective miRNAs such as miR-425 (Wu Y. et al., 2021). Because natural EVs contain heterogeneous cargoes, individual vesicles may carry both protective and detrimental components, potentially leading to variable outcomes. To overcome this limitation, engineered synthetic vesicles have been proposed as targeted delivery systems. By selectively loading protective miRNAs, such as let-7a-5p, and administering them via inhalation, these vesicles may reduce EVs heterogeneity and enable more precise therapeutic strategies for HALI (; Chen et al., 2024; Ravikumar et al., 2016; Wu et al., 2017). However, their clinical translation still requires evaluation of cargo stability, biodistribution, immunogenicity, and off-target effects.

Overall, cell and molecule based therapies show promise in experimental HALI, but most evidence remains limited to cell and animal models. Their long-term safety, reproducibility, and clinical efficacy remain unclear, highlighting the need for further translational and clinical studies (The therapeutic interventions, major targets, mechanisms and protective effects are summarized in Table 2)

**TABLE 2:** Therapeutic strategies, major mechanisms and protective effects in HALI.

Therapeutic category		Interventions	Targets/mechanisms	Protective effects
Oxidative stress regulation	Antioxidants	Etomidate, lycium barbarum polysaccharides, wedelolactone, sulforaphane, melanin nanodots, sEH inhibition	↑NRF2/HO-1; ↑endogenous antioxidant; ↓ROS, M1 macrophage	↓Oxidative stress, inflammation ↓apoptosis and ferroptosis
	CYP1A inducers	BNF	↑CYP1A1/2; ↑xenobiotic metabolism and detoxification	↓Oxidative stress and inflammation
	Iron modulators	Lactoferrin, DFO	↓Iron, ROS and lipid peroxidation	↓Iron accumulation, oxidative injury and ferroptosis
	Anti-ferroptotic agents	NMN	↑GPX4	↓Ferroptosis
Anti-inflammatory and immunomodulatory strategies	—	Ascorbic acid, sulforaphane; GTS-21, probucol; astragaloside, aspirin; salidroside, simvastatin, roflumilast, IL-10, SOCS-1	↓HMGB1, NF-κB; ↑p38 MAPK, cAMP	↓Pro-inflammatory cytokine production; ↓macrophage/neutrophil activation and infiltration
Metabolic and gut–lung axis regulation	—	High-fiber diet, acetate supplementation	Modulation of gut microbiota; ↑short-chain fatty acid production;	↑Beneficial microbial metabolites; ↓inflammation and oxidative stress
Mitochondrial function regulation	—	Alda-1, mitoQ, 10-nitrooleic acid, stanniocalcin-1, resveratrol, thioredoxin	↑ALDH2, SIRT1, p38MAPK, PGC-1α, UCP2	Maintenance of MMP and energy metabolism; ↓ROS, apoptosis
Alveolar barrier protection	—	4-PBA, B-YL + PGZ, exogenous surfactant + N-acetylcysteine	↑Claudin-4, PPARγ; ↓ERs, NF-κB, Wnt/TGF-β signaling	↑AECs barrier integrity; ↓pulmonary edema, inflammation and apoptosis
Gas molecule therapy	—	Hydrogen, inhaled NO	↑SIRT1; ↓PAI-1	↓ERs,apoptosis, fibrin deposition and edema
Cell and molecular therapy	Stem cell therapy	iPSCs, ALMs	↓oxidative stress, inflammation, Src/FOXO1	↑lung repair; preservation of pulmonary immune and antibacterial functions
	EVs and miRNAs	EVs, miR-425, miR-20b, miR-21–5p, miR-16, let-7a-5p, miR-185 inhibition	↓MFN1/2, TGF-β/Smad2, JAK/STAT3; ↑PTEN/AKT	↓Apoptosis, mitochondrial dysfunction; targeted delivery of protective molecules

## 4 Current limitations and future perspectives

Although multiple gene targets and signaling pathways involved in HALI have been identified, the critical regulators that determine the initiation, progression, and resolution of injury remain unclear. Given the concentration- and time-dependent nature of HALI, future studies should characterize the spatiotemporal dynamics of molecular responses rather than relying on single time-point measurements. Stage-specific regulation may provide new therapeutic opportunities. for example, suppressing miR-185 during the early injury phase (Zhang D. et al., 2016) or activating miR-21 during the repair phase (Qin et al., 2019). In addition, identifying key nodes that drive the progression from acute injury to pulmonary fibrosis is essential for developing strategies that prevent early damage and inhibit chronic remodeling. Elucidating intercellular communication among AMs, AECs, and ECs will also be important because HALI cannot be fully explained by changes in a single cell population. Future studies could integrate single-cell RNA sequencing, spatial transcriptomics, ligand–receptor interaction analysis, and lineage-tracing approaches to define cell-specific responses and intercellular communication during hyperoxia. Advanced models, including AEC–macrophage/EC co-culture systems, lung organoids, and lung-on-a-chip platforms, could further reproduce the alveolar–capillary interface and controlled hyperoxic exposure. Combining these models with live-cell imaging and CRISPR-based perturbation screening may help identify causal regulatory nodes and distinguish therapeutic targets from secondary responses.

Despite extensive experimental research, clinically relevant evidence for the molecular mechanisms of HALI remains limited. Most mechanistic studies have relied on rodent models or isolated lung epithelial cells, which cannot fully reproduce the heterogeneity, comorbidities, oxygen exposure patterns, and treatment interventions encountered in critically ill patients. Moreover, the lack of serial human lung tissue samples makes it difficult to determine whether molecular changes identified in experimental models represent causal events or secondary responses. Future multicenter studies should therefore integrate clinical phenotypes with high-throughput analyses of bronchoalveolar lavage fluid, sputum, blood, and, when available, lung tissue. Longitudinal sampling may be particularly valuable for identifying molecular changes associated with injury initiation, progression, and recovery. Such efforts will help establish diagnostic and prognostic models and support precision medicine strategies for HALI.

Therapeutic translation represents another major limitation. Most candidate interventions reviewed in this article have demonstrated efficacy primarily in cell or animal models, whereas their clinical effectiveness remains uncertain. Differences in oxygen concentration, exposure duration, disease background, treatment timing, dosage, and administration route may contribute to discrepancies between experimental models and clinical settings. Even clinically available agents, such as aspirin (Tung et al., 2021) and Rhodiola rosea–derived compounds (Guo et al., 2022), require clinical evaluation before their protective effects in experimental HALI can be extrapolated to patients. Emerging biological therapies face additional translational barriers. Stem cell therapy has shown potential to attenuate inflammation and oxidative stress and promote tissue repair; however, limited cell survival and engraftment, heterogeneity of cell preparations, immune compatibility, manufacturing and storage requirements, and potential tumorigenicity remain important concerns. Similarly, gene-editing approaches such as CRISPR–Cas9 provide powerful tools for validating disease-driving genes, but therapeutic application requires rigorous assessment of off-target effects, delivery efficiency, tissue specificity, immunogenicity, and long-term safety. EV-based therapies may avoid some limitations of cell transplantation, but variability in cargo composition, production, purification, storage, and biodistribution may affect their reproducibility. Engineered EVs and targeted nanoparticle systems may improve cargo delivery (Han et al., 2025), but their long-term safety, immunogenicity, pulmonary retention, and manufacturing reproducibility require systematic evaluation before clinical application.

The extensive crosstalk among oxidative stress, inflammation, organelle dysfunction, and regulated cell death also highlights the limitations of single-target therapies. Many antioxidants, for example, regulate both oxidative stress and inflammatory responses (Jia et al., 2021), indicating that multi-target strategies may be more effective. For instance, combining NLRP3 inflammasome inhibitors with ferroptosis inhibitors could simultaneously modulate inflammatory and cell death pathways. By incorporating organ-specific targeting ligands and delivering anti-inflammatory mediators such as IL-10 or protective compounds, these strategies may enhance efficacy while minimizing complications associated with EV-based therapies, ultimately enabling safer and more precise interventions for HALI. However, multi-target intervention is not necessarily superior, excessive suppression of inflammatory responses or cell death pathways may interfere with host defense or tissue repair. Future studies should therefore determine the optimal treatment sequence, therapeutic window, and patient subgroup rather than simply increasing the number of therapeutic targets.
